# Exosomal miR-493 suppresses MAD2L1 and induces chemoresistance to intraperitoneal paclitaxel therapy in gastric cancer patients with peritoneal metastasis

**DOI:** 10.1038/s41598-024-60967-x

**Published:** 2024-05-02

**Authors:** Masahiro Makinoya, Kozo Miyatani, Yoshiaki Matsumi, Yu Sakano, Shota Shimizu, Yuji Shishido, Takehiko Hanaki, Kyoichi Kihara, Tomoyuki Matsunaga, Manabu Yamamoto, Naruo Tokuyasu, Shuichi Takano, Teruhisa Sakamoto, Toshimichi Hasegawa, Hiroaki Saito, Yuji Nakayama, Mitsuhiko Osaki, Futoshi Okada, Yoshiyuki Fujiwara

**Affiliations:** 1 Division of Gastrointestinal and Pediatric Surgery, Department of Surgery, School of Medicine, Tottori University Faculty of Medicine, 36-1 Nishi-cho, Yonago, 683-8504 Japan; 2 Division of Chemical Biology, Technical Department, Tottori University, 36-1 Nishi-cho, Yonago, 683-8504 Japan; 3 Department of Surgery, Japanese Red Cross Tottori Hospital, Tottori, 680‑8517 Japan; 4 Division of Radioisotope Science, Research Initiative Center, Organization for Research Initiative and Promotion, Tottori University, 36-1 Nishi-cho, Yonago, 683-8504 Japan; 5 Division of Experimental Pathology, Faculty of Medicine, Tottori University, 36-1 Nishi-cho, Yonago, 683-8504 Japan; 6 Chromosomal Engineering Research Center, Tottori University, 36-1 Nishi-cho, Yonago, 683-8504 Japan

**Keywords:** Cancer, Gastroenterology, Oncology

## Abstract

Intraperitoneal (IP) chemotherapy with paclitaxel (PTX) for gastric cancer (GC) with peritoneal metastasis (PM) is considered a promising treatment approach, however, there are no useful biomarkers to predict the efficacy of IP therapy. We examined the association between intra-peritoneal exosomes, particularly exosomal micro-RNAs (exo-miRNAs), and IP-chemo sensitivity. MKN45 cells that were cultured with intra-peritoneal exosomes from patients who did not respond to IP therapy with PTX (IP^non-respond^ group) exhibited resistance to PTX compared with exosomes from responding patients (IP^respond^ group) (*p* = 0.002). A comprehensive search for exo-miRNAs indicated that miR-493 was significantly up-regulated in exosomes from the IP^non-respond^ group compared with those collected from the IP^respond^ group. The expression of miR-493 in PTX-resistant MKN45 cells (MKN45^PTX-res^) was higher compared with that in MKN45. In addition, MKN45^PTX-res^ cells exhibited lower MAD2L1 gene and protein expression compared with MKN45. Finally, miR-493 enhancement by transfection of miR-493 mimics significantly down-regulated MAD2L1 expression in MKN45 cells and reduced PTX sensitivity. Our results suggest that intra-peritoneal exo-miR-493 is involved in chemoresistance to PTX by downregulating MAD2L1 in GC with PM. Exo-miR-493 may be a biomarker for chemoresistance and prognosis of GC patients with PM and may also be a promising therapeutic target.

## Introduction

Gastric cancer (GC) ranks as the sixth most common cancer and the third leading cause of cancer-related deaths worldwide^1^. The morbidity and mortality rates of GC have been declining^2,3^, possibly because of the widespread eradication of *Helicobacter pylori* and early detection with improved endoscopic diagnostic techniques. However, the prognosis of advanced GC with serosal involvement remains poor^4^, and even if gastrectomy can be performed, approximately 20% of the cases develop peritoneal recurrences^5^. In addition, the peritoneum is the most frequent site of distant metastasis in GC^6^ and PM has a significant negative impact on the prognosis of advanced GC patients^4^. Advanced GC patients with PM are usually treated with a combination of platinum agents plus fluoropyrimidine, with or without trastuzumab, depending on HER2 status as first-line treatment^7–9^. More recently, immune checkpoint inhibitors (ICIs) in combination with first-line chemotherapy have been approved as standard chemotherapy and are used for unresectable advanced or recurrent GC, including cases of PM^10,11^. Despite these advances in systemic chemotherapy, the prognosis of GC patients with PM remains unsatisfactory. Alternatively, the PHOENIX-GC Trial indicated that IP chemotherapy with paclitaxel (PTX) was safe and effective for GC patients with PM^12^. Currently, the PHOENIX-GC2 trial is underway to verify the efficacy of IP chemotherapy with PTX to prevent postoperative peritoneal recurrence in GC patients with Type 4 tumor^13^. Thus, IP chemotherapy has attracted significant attention, although the identification of biomarkers to predict the treatment efficacy of IP therapy with PTX is needed.

Exosomes are small phospholipid bilayer vesicles of approximately 100 nM in diameter that are used for cell-to-cell communication by transporting functional molecules, such as proteins, mRNAs, and miRNAs^14^. miRNAs are non-coding RNAs consisting of 22–25 base pairs that bind to the untranslated region of the target mRNA and mediate its inhibition of translation^15^. Moreover, recent studies have found that some exosomes play an important role in tumor progression. Shibamoto et al. demonstrated that the removal of GC-derived exosomes resulted in the suppression of the proliferation and migration ability of GC cells and the adhesion of GC cells to peritoneal mesothelial cells, suggesting that exosomes were involved in the progression of PM^16^. Among the exosome inclusions, exo-miRNAs are considered one of the most abundant and stable molecules, which escape enzymatic degradation by RNase and influence cancer progression, proliferation, and chemotherapy resistance^17^. Recently, studies on the clinical application of exo-miRNAs as biomarkers for early diagnosis and tumor progression are an active area of investigation^18,19^.

With respect to exo-miRNAs for the treatment of GC with PM, Ohzawa et al. showed that the expression of exo-miRNAs in peritoneal fluid before treatment may be a useful biomarker to determine the existence of PM and to predict the postoperative peritoneal recurrence^20^. Furthermore, to our knowledge, only one study demonstrated the involvement of exo-miRNAs in the acquisition of drug resistance to IP chemotherapy^21^. Therefore, we identified novel exo-miRNAs involved in the development of drug resistance and elucidated the mechanisms involved in the increased chemoresistance to IP chemotherapy with PTX.

## Methods

### Patient samples and tumor specimens

Fifty patients were enrolled with a histopathological diagnosis of gastric adenocarcinoma and treated at Tottori University Hospital. Of these, exosomes were collected from the peritoneal lavage fluid of four patients who underwent staging laparoscopy (SL) and had pathologically proven PM. They were treated with IP and intravenous (IV) PTX combined with oral S-1 chemotherapy. Exosomes from the peritoneal lavage fluid of one GC patient without PM were also collected to use for quantitative real-time PCR (qRT-PCR). The IP therapy regimen was based on the PHOENIX-GC Trial^12^. To prepare patients for IP PTX therapy, an access port (BARD port-Ti; Becton, Dickinson and Company, NJ, USA) was subcutaneously implanted during staging laparoscopy. This treatment was performed as a clinical study approved by the Institutional Review Board of Tottori University (approval number: C1704B011). Written informed consent was obtained from all patients. Tissue samples were immunohistochemically stained for 45 patients who experienced a recurrence after gastrectomy and received PTX-based chemotherapy at Tottori University Hospital from January 2006 to December 2019. The immunohistochemistry experiment was approved by the Institutional Review Board of Tottori University (approval number: 20A137). In addition, all methods were performed in accordance with the relevant guidelines and regulations.

### Extracellular vehicles extraction

**Figure 1:**
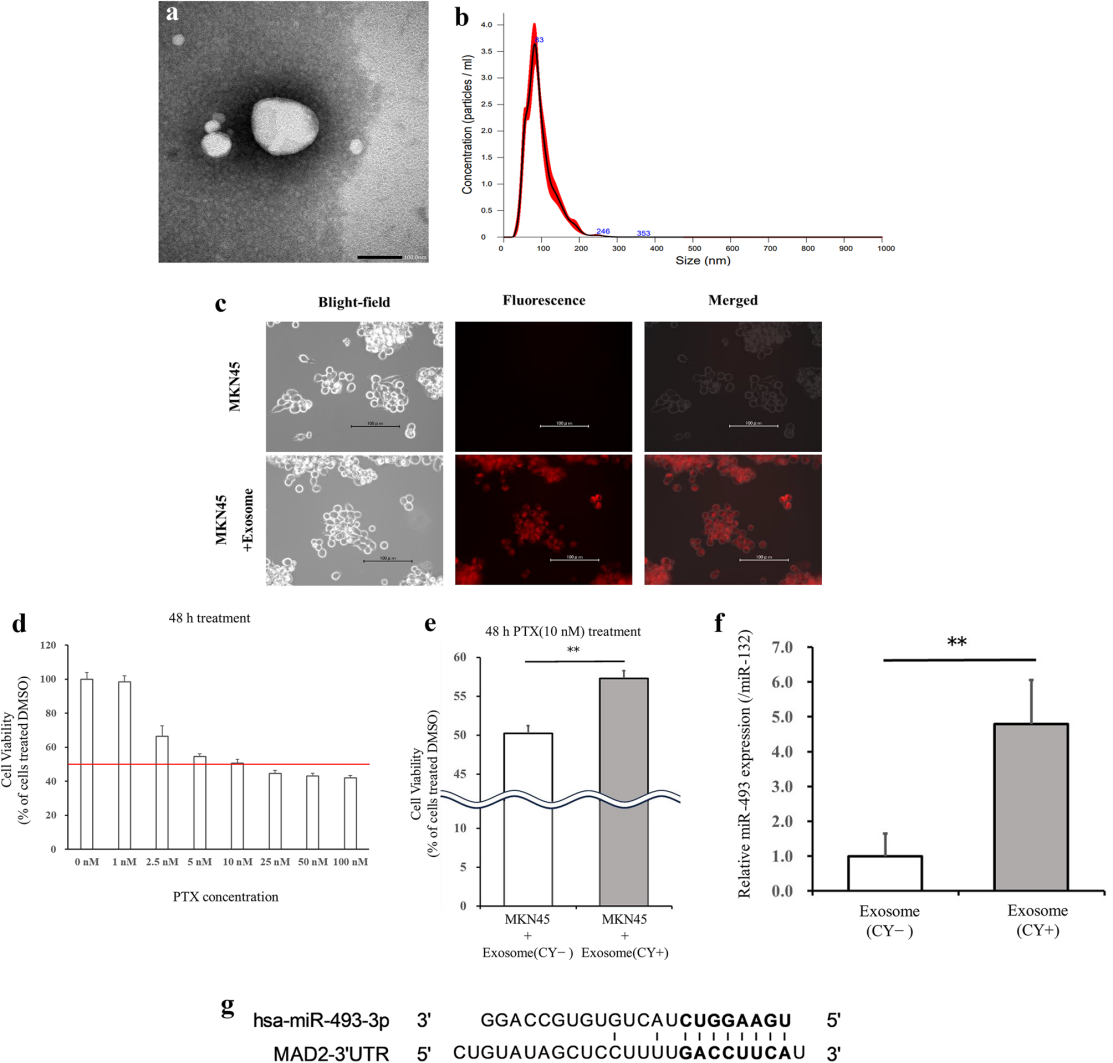
Characterization of exosomes extracted from the peritoneal fluid of GC patients with PM. (**a**) Representative image of exosomes detected using transmission electron microscopy. Scale bar: 100 nm. (**b**) The concentration and size distribution of exosomes were analyzed using NanoSight NS300. (**c**) The uptake of labeled exosomes after 24 h of incubation was confirmed by fluorescence microscopy. Scale bar: 100 μm. (**d**) The IC_50_ of MKN45 to PTX was measured using the WST assay. (**e**) Comparison of the sensitivity of MKN45 cells cocultured with CY + and CY − group exosomes to PTX. (**f**) Comparison of miR-493 expression in exosomes between the CY + and CY − groups. Statistical differences were determined using a Student’s *t*-test (*p < 0.05, **p < 0.005, ***p < 0.001). (**g**) Schematic illustration of the predicted target site of miR-493-3p in the Mad2 mRNA 3’UTR.

Peritoneal lavage fluid was collected at the time of SL and the beginning of each cycle of chemotherapy. At the beginning of SL, 100 mL of normal saline was injected into the rectouterine pouch and peritoneal lavage fluid was collected. At the beginning of every cycle of chemotherapy, 400 mL of normal saline was introduced into the peritoneal cavity through the IP access port and approximately 50 mL of peritoneal lavage fluid was collected. A portion of the peritoneal lavage fluid was submitted for cytology, whereas the remainder was centrifuged at 3000×*g* for 15 min to remove impurities. The extracellular vehicles (EVs) were isolated by the ExoQuick exosome isolation kit (RA820A-1; System Biosciences, LLC, CA, USA). The extracted EVs were evaluated using a JEM-1400 transmission electron microscope (JEOL Ltd., Tokyo, Japan) (Fig. 1a) and NanoSight NS300 (Malvern Panalytical, Malvern, England) for nano-tracking analysis (Fig. 1b). The major population of EVs had a diameter of approximately 100 nM.

### Cell culture

The human GC cell line MKN-45 was purchased from the Riken Cell Bank (Tsukuba, Japan) and cultured in Roswell Park Memorial Institute medium (RPMI 1640; FUJIFILM Wako Pure Chemical Co., Osaka, Japan) supplemented with 10% fetal bovine serum (Cosmo Bio Co., Ltd, Tokyo, Japan) and 1% penicillin–streptomycin solution (FUJIFILM Wako Pure Chemical Co.). The cells were maintained at 37 °C in atmospheric air supplemented with 5% carbon dioxide (CO_2_) and passaged at a ratio of 1:3–1:20 every 3–7 days.

### Labeling of exosomes and fluorescence microscopy

For in vitro uptake studies, the purified exosomes were fluorescently labeled using the ExoGlow™-Membrane EV Labeling Kit (EXOGM600A-1; System Biosciences, LLC) based on the manufacturer’s instructions. MKN45 cells were seeded into glass bottom dishes and cultured with the labeled exosomes at 37 °C overnight. After washing with conditioned medium, exosome uptake by MKN45 cells was confirmed using fluorescence microscopy (BZ-9000; KEYENCE CORPORATION, Osaka, Japan). The excitation and emission wavelengths were 465 nm and 635 nm, respectively.

### Cell proliferation assay

MKN45 cell viability was determined using the WST assay. Cells were seeded at 1.5 × 10^4^ per well in 96-well plates and incubated overnight at 37˚C in a humidified incubator containing 5% CO_2_. Paclitaxel (Selleck Chemicals, TX, USA) was dissolved in DMSO (D2650, Sigma-Aldrich, MO, USA) and added to the cell culture medium at various concentrations (0–100 nM). After a 48-h incubation, 10% (v/v) of the Cell Counting Kit-8 solution (DOJINDO, Kumamoto, Japan) was added to each well and incubated at 37 ℃ for 1 h. The absorbance was measured using an Infinite F50 microplate reader (TECAN, Kawasaki, Japan) at 450nm and at a reference wavelength of 620 nm to assess cell proliferation. Cell viability was calculated as follows: viability = (absorbance of experimental wells)/(absorbance of control wells, respectively). The values represent means ± SD of five separate wells. The IC_50_ was calculated as the PTX concentration that inhibited 50% cell viability.

### RNA extraction

Nano-drop (Thermo Fisher Scientific, MA, USA) was used to measure RNA concentration. Exo-miRNAs were extracted from the EVs using the Exosome RNA Amplification and Profiling Complete Kit (RA820A-1; System Biosciences, LLC). Total RNA extraction and reverse transcription were done as previously described^22^. Briefly, MKN45 cells were seeded in 6-well plates containing three independent samples per condition. Total RNA was isolated using TRI reagent (Molecular Research Center, Inc., OH, USA) and the PureLink RNA Mini Kit (Thermo Fisher Scientific).

### Reverse transcription and qRT-PCR analysis

A thermal cycler (Hangzhou Bioer Technology Co. Ltd., Hangzhou, PRC) was used for reverse transcription and qRT-PCR was done using the ViiA7 system (Applied Biosystems, CA, USA). Exo-miRNA expression was analyzed using Plate#1 Human MicroRNA qPCR Primers (RA820A-1) and the Maxima SYBR Green/ROX qPCR Master Mix (2X) (Thermo Fisher Scientific). For quantitating individual miRNAs, TaqMan miRNA Assays (miR-132, miR-99a, miR-193a-5p, miR-300, miR-362, miR-493, miR-500 and miR-532, RUN6B) (#4427975; Thermo Fisher Scientific) and TaqMan Fast Advanced Master Mix (Thermo Fisher Scientific) were used. For targeting gene-specific mRNA in cancer cells, cDNA was synthesized using SuperScript IV VILO Master Mix (Thermo Fisher Scientific). MAD2L1 (Hs01554513_g1; Thermo Fisher Scientific) and ACTB (Hs99999903_m1; Santa Cruz Biotechnology, TX, USA) primers along with TaqMan Fast Advanced Master Mix were used for qPCR. The results were analyzed using a Student’s t-test and expressed by standard deviation. The values represent means ± SD of 3–5 separate wells.

### Identification of exo-miRNAs

The NormFinder algorithm^23^ was used to identify the most stably expressed miRNAs based on the results of a comprehensive search of exo-miRNA expression. Quantitation was performed using the ΔCT method. Ct values were normalized to the miRNAs selected from the NormFinder algorithm. Exo-miRNA samples extracted from peritoneal lavage fluid were divided into two groups based on whether peritoneal cytology (CY) was positive or negative within three courses of IP chemotherapy. The miRNAs with a fold-change < 0.33 were considered down-regulated and miRNAs with an expression > 1.5 were considered up-regulated.

### Western blot analysis

Cells were lysed in RIPA buffer (Nacalai Tesque, Kyoto, Japan) and centrifuged at 15,000*rpm* for 10 min at 4 °C. The resulting supernatants were collected and the protein concentration was determined using the Bradford Protein Assay (Takara Bio Inc., Shiga, Japan). The proteins were separated on 12% Mini-PROTEAN TGX Precast Gels (Bio-Rad, CA, USA) and transferred to 0.2-µm polyvinylidene difluoride membranes (Bio-Rad). After blocking for 1 h at room temperature with ECL Prime Blocking Agent (Cytiva, Tokyo, Japan), the membranes were incubated with a primary antibody against MAD2L1 (1:500) (#10337-1-AP; Proteintech Group, Inc, IL, USA) for 16 h at 4 °C. A primary antibody against β-actin (1:2000) (Santa Cruz Biotechnology) was used for normalization. After washing, mouse and rabbit peroxidase-linked secondary antibodies (both from Cytiva) were incubated with the membranes for 1 h at room temperature. The protein signals were detected using an ECL Prime Western blotting detection reagent (Cytiva) and quantified using ImageQuant 500 (Cytiva). The intensity of the protein bands was compared using ImageJ software and the experiments were performed in triplicate.

### Transient transfection and PTX sensitivity assay

Silencer Select small interference RNA (siRNA) targeting the MAD2L1 gene (s8392; Thermo Fisher Scientific), a non-silencing siRNA control (sc-37007; Santa Cruz Biotechnology), has-miR-493 mirVana™ miRNA mimic (4464066; Thermo Fisher Scientific), mirVana™ miRNA mimic Negative Control #1 (4464058; Thermo Fisher Scientific), has-miR-493 mirVana™ miRNA inhibitor (4464084; Thermo Fisher Scientific), and mirVana™ miRNA Inhibitor, Negative Control #1 (4464076; Thermo Fisher Scientific) were purchased. Cells were seeded into either 6- or 96-well plates at densities of 1.5 × 10^5^ cells/mL and transfected with Lipofectamine RNAiMAX (Thermo Fisher Scientific). Silencer Select siRNA, Opti-MEM (Invitrogen, CA, USA), and Lipofectamine RNAiMAX transfection reagents were mixed and incubated for 5 min at room temperature. The siRNA-lipid complex was added to each well. After 72 h, the effect of mRNA silencing was confirmed by qPCR and western blot analysis. To evaluate the sensitivity of the cells to PTX, the medium was replaced 6 h after siRNA transfection. After 24 h, the cells were treated with PTX or DMSO for 48 h and cell viability was determined by the WST assay. Values represent means ± SD of three separate wells. Antibiotics were not added to the growth medium during this experiment.

### Immunohistochemical analysis

Paraffin-embedded specimens were prepared for immunohistochemical analysis, which was performed according to standard protocols. Briefly, formalin-fixed, paraffin-embedded tissues were processed into 4 μm-thick sections. After deparaffinizing, endogenous peroxidase was blocked by incubation with 3% hydrogen peroxide for 30 min and non-specific protein binding was blocked with 1% Block-Ace (DS Pharma Biomedical, Osaka, Japan) for 30 min. Primary antibody against MAD2L1 (10337-1-AP, Proteintech Group, Inc) was used at a 1:400 dilution. Specimens were incubated with primary antibody overnight at 4 °C. The slides were incubated with MAX-PO (MULTI) (NICHIREI BIOSCIENCES INC, Tokyo, Japan) followed by secondary antibody. Color development was done using a DAB Peroxidase Substrate Kit (Vector Laboratories, Burlingame, CA, USA) and the sections were counterstained with hematoxylin. MAD2L1 expression in GC cells was assessed on a 4-point scale based on the percentage of tumor cells exhibiting positive staining (immunohistochemistry score: 0 [0–24%], 1 [25–49%], 2 [50–74%], 3 [75–100%]). A score ≥ 2 was considered high expression. Immunolabeling was evaluated by two investigators (Y.U., M.M.) and a consensus was reached in all cases. Patients were classified as having a CR (complete response), PR (partial response), SD (stable disease), PD (progressive disease), or NE (not evaluable). ORR was defined as the percentage of patients with either a CR or PR. The disease control rate (DCR) was defined as the percentage of patients with either a CR, PR, or SD.

### Survival analysis

Survival curves were estimated by a Kaplan–Meier analysis. Differences in survival curves were compared using the log-rank test. Cox proportional hazard models were used for univariate and multivariate analyses. All quantitative values are presented as the median. Overall survival was defined as the time from PTX initiation to death from any cause. Relapse-free survival was defined as the time from PTX initiation to the occurrence of an event, relapse or death, whichever occurred first. Data for patients who had not had an event were censored as of the date of the final observation. The association between MAD2L1 expression and patient characteristics was examined using a chi-squared test and Fisher’s exact test for categorical variables. All p values < 0.05 were considered statistically significant. Statistical analyses were performed using SPSS version 27.0 software (IBM, NY, USA).

### Establishment of a PTX-resistant MKN45 cell line

Resistant strains were generated based on prior papers^24^. Specifically, PTX-resistant MKN45 (MKN45^PTX-res^) cells were established by exposure to low, increasing concentrations of PTX. MKN45 cells were first exposed to 0.1 nM PTX. Once they reached 80% confluence, they were passaged. After two passages at the same concentration, the PTX concentration was increased slightly and passed in the same manner. This procedure was repeated until the IC_50_ was more than three times the IC_50_ of the parental strain. The resistance index (RI) was calculated as the IC_50_ of MKN45^PTX-res^/IC_50_ of MKN45. The RI was greater than three-fold higher compared with the previous strain, which indicated that a resistant strain had been established.

### Cell cycle analysis by flow cytometry

MKN45 and MKN45^PTX-res^ were treated as follows: normal medium, 16 h PTX treatment, and 48 h PTX treatment. To analyze DNA content, the cells were collected by trypsinization, fixed in D-PBS (FUJIFILM Wako Pure Chemical Co.) at room temperature with ice-cold 70% ethanol/30% PBS, and stored at – 30 °C. After centrifugation, the supernatant was discarded, the cells were washed twice with cold PBS, and incubated with 0.25 mg/mL RNase A (NIPPON GENE CO., LTD., Tokyo, Japan) at 37 °C for 30 min. The cells were then stained with propidium iodide. Flow cytometric analyses were performed using a BD LSRFortessa™ X-20 (Becton–Dickinson and Company) and the sub-G0/G1, S, and G2/M phase fractions of 1 × 10^6^ cells were determined by flow cytometry.

## Results

### Relationship between exosomes and acquisition of PTX chemoresistance

We determined whether exosomes derived from the peritoneal fluid of GC patients with PM were associated with the acquisition of PTX resistance in the MKN45 GC cell line. To confirm the uptake of exosomes into MKN45 cells, the exosomes labeled with the ExoGlow-Membrane kit were co-cultured with MKN45 cells. Localization of the exosomes in MKN45 cells was confirmed, indicating cellular uptake of the co-cultured exosomes (Fig. 1c). Four patients with CY-positive peritoneal fluid who received IP chemotherapy were divided into two groups based on whether CY became negative (CY − group) or remained positive (CY + group) during three courses of IP chemotherapy. Prior to PTX resistance analysis, we confirmed the IC_50_ of PTX in MKN45 cells (Fig. 1d) and set the concentration of PTX for subsequent experiments at 10 nM. Cells treated with exosomes from the CY + group were resistant to PTX compared with those treated with exosomes from the CY − group (*p* = 0.002, Fig. 1e). These results indicate that exosomes derived from the peritoneal fluid of GC patients conferred PTX chemoresistance in MKN45 cells.

### Selection of candidate miRNAs associated with PTX chemoresistance

MiRNAs have been reported to influence cancer progression, proliferation, and chemotherapy resistance. We determined the effect of exo-miRNAs on acquired resistance to IP PTX. A comprehensive search for exosomal miRNAs was performed by evaluating the expression of 380 miRNAs using the Exosome RNA Amplification and Profiling Complete Kit. The resulting Ct values were normalized using the NormFinder algorithm. NormFinder selected miR-132 as the normalizer, which was used in subsequent experiments to correct for exo-miRNA expression in peritoneal lavage fluid. Four miRNAs (miR-362-5p, miR-493, miR-500, and miR-523) were identified as up-regulated miRNAs and two (miR-99a and miR-193a-5p) were down-regulated in the CY + group compared with the CY − group. These six miRNAs were associated with the acquisition of chemoresistance to IP PTX. To narrow down the candidate miRNAs, their expression was validated in all 6 samples using TaqMan miRNA assays. miR-493 exhibited a most significant difference in expression between the CY + and CY − groups (*p* = 0.002, Fig. 1f, Supplementary Fig. S1). The results indicated that exosomal miR-493 exacerbates PTX chemosensitivity.

### MAD2L1 expression predicts the prognosis of recurrent GC patients treated with PTX-based chemotherapy

**Figure 2:**
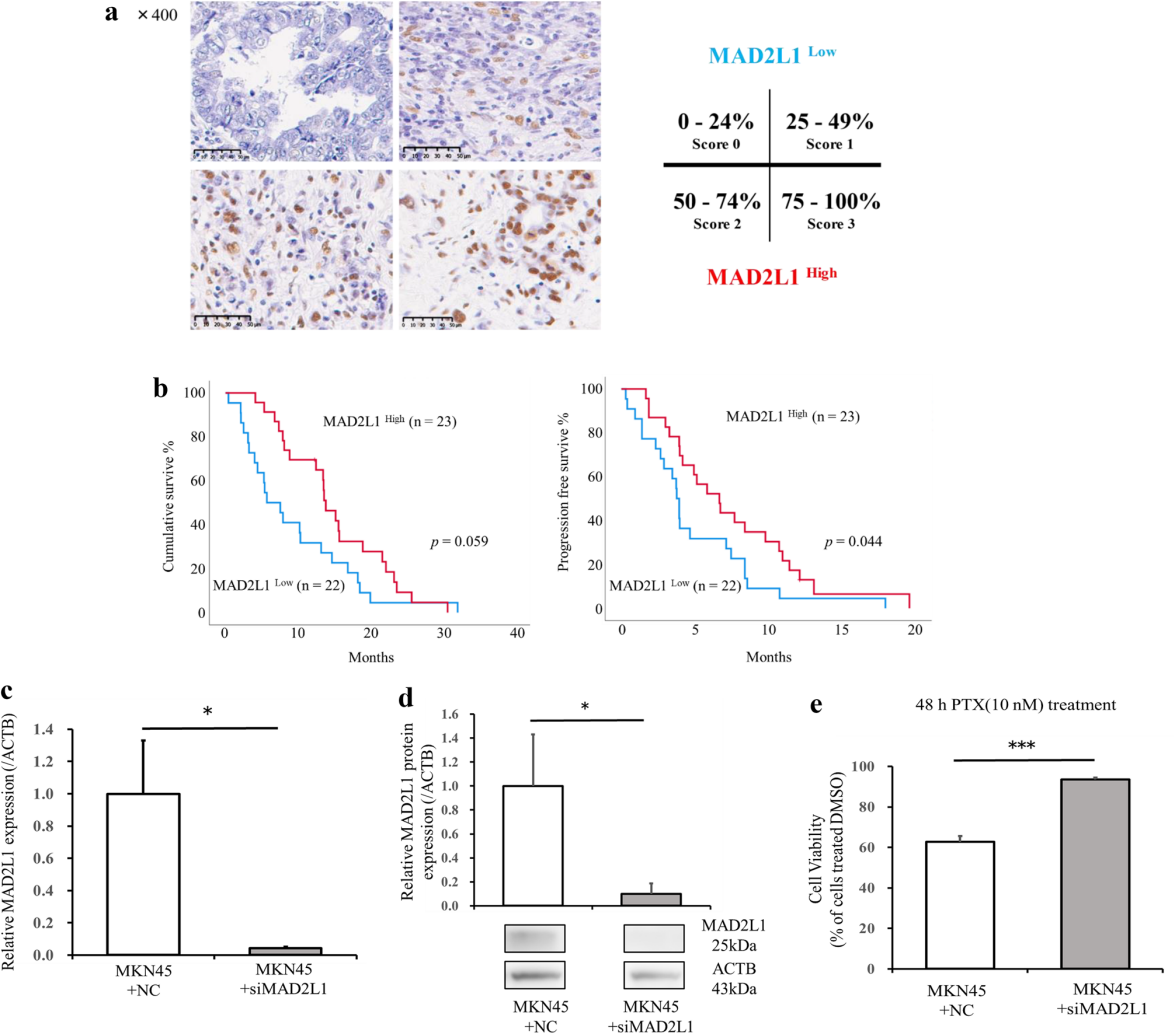
MAD2L1 expression in GC tissues predicts the prognosis of recurrent GC patients treated with PTX-based chemotherapy. (**a**) Representative images of GC tissue samples stained for MAD2L1 by immunohistochemistry. Scale bar: 50 μm. (**b**) Overall survival (left) and progression-free survival (right) of recurrent GC patients treated with PTX-based chemotherapy. Survival rates were calculated using the Kaplan–Meier method and differences were evaluated using a log‐rank test. (**c**) MAD2L1 gene and (**d**) protein expression levels in MKN45 following siRNA knockdown of MAD2L1. Statistical differences were determined using a Student’s *t*-test (*p < 0.05, **p < 0.005, ***p < 0.001). (**e**) Comparison of the sensitivity of siMAD2L1-treated MKN45 cells and control-treated MKN45 cells to PTX. Viability was quantified using the CCK-8 assay after a 48-h treatment. The results are presented as a percentage of viable cells relative to cells treated with DMSO. Data show the results from five independent experiments, performed in quadruplicate. Statistical differences were determined using a Student’s *t* test (*p < 0.05, **p < 0.005, ***p < 0.001).

A search of the miRBase (https://mirbase.org/) yielded reports on MAD2L1-mediated decreased susceptibility of ovarian cancer to PTX by miR-493^25^ (Fig. 1g). We considered the possibility of a similar event occurring in gastric cancer. Therefore, we examined MAD2L1 expression in the tissues of GC patients who experienced a recurrence after gastrectomy and were treated with PTX-based systemic chemotherapy. The association between MAD2L1 expression and chemosensitivity to PTX chemotherapy was determined. An immunohistochemical analysis revealed that MAD2L1-positive staining was predominantly observed in the nucleus of the cancer cells in GC tissue (Fig. 2a). We found that 22 of 45 patients (48.9%) exhibited low MAD2L1 expression. There was no correlation between MAD2L1 expression and each of the clinicopathological factors examined (Table 1). The overall survival (OS) rate tended to be decreased in MAD2L1-low patients (MAD2L1^Low^ group) compared with MAD2L1-high patients (MAD2L1^High^ group) (*p* = 0.059). Furthermore, progression free survival (PFS) was significantly lower in the MAD2L1^Low^ group compared with the MAD2L1^High^ group (*p* = 0.044) (Fig. 2b). Analysis of the relationship between MAD2L1 expression and chemo-sensitivity revealed that the overall response rate was significantly lower in the MAD2L1^Low^ group compared with the MAD2L1^High^ group (*p* = 0.043) (Table 2).

**Table 1:** Association between MAD2L1 expression and clinicopathological factors.

Characteristics	MAD2L1 expression, *n* (%)		p-value
	High	Low	
	*n* = 23	*n* = 22	
Age, years			0.284
< 75	16 (69.6)	19 (86.4)	
≥ 75	7 (30.4)	3 (13.6)	
Gender			0.233
Male	20 (87.0)	16 (72.7)	
Female	3 (13.0)	6 (27.3)	
Differentiation			0.300
Differentiated	14 (60.9)	8 (36.4)	
Poorly differentiated	9 (39.1)	14 (63.6)	
Depth of invasion^a^			0.242
pT1	5 (21.7)	2 (9.1)	
pT2/3/4	18 (78.3)	20 (90.9)	
Lymph node metastasis			0.953
Absent	3 (13.0)	3 (13.6)	
Present	20 (87.0)	19 (86.4)	
Distant metastasis			0.396
Absent	20 (87.0)	17 (77.3)	
Present	3 (13.0)	5 (22.7)	
Number of chemotherapy line			0.231
1st line	12 (52.2)	7 (31.8)	
≧ 2nd line	11 (47.8)	15 (68.2)	

^a^Depth of invasion: T1, tumor invasion of lamina propria or submucosa; T2, tumor invasion of muscularis propria or subserosa; T3, tumor penetration of serosa; T4, tumor invasion of adjacent organs.

**Table 2:** Association between MAD2L1 expression and treatment response determination.

Item, *n* (%)	Effectiveness analysis set		p-value
	MDA2L1		
	High (*n* = 23)	Low (*n* = 22)	
CR	1 (4.4)	0 (0.0)	
PR	5 (21.7)	3 (13.6)	
SD	10 (43.5)	9 (40.9)	
PD	7 (30.4)	5 (22.7)	
NE	0 (0.0)	5 (22.7)	
ORR^a^	6 (26.1)	3 (13.6)	0.043*

*CR* complete response, *PR* partial response, *SD* stable disease, *PD* progressive disease, *NE* not evaluable, *ORR* overall response rate.

^a^CR + PR.

*p < 0.05.

### MAD2L1 regulates PTX chemosensitivity in MKN45 cells

To confirm whether MAD2L1 regulates PTX chemosensitivity, we determined the effect of downregulating MAD2L1 expression in MKN45 cells following targeted siRNA transfection. The siRNA targeting MAD2L1 efficiently reduced MAD2L1 gene (Fig. 2c; *p* = 0.007) and protein (Fig. 2d, Supplementary Fig. S2; *p* = 0.024) expression in MKN45 cells. Moreover, MKN45 cells treated with siMAD2L1 were less sensitive to PTX compared with the control cells (*p* < 0.001, Fig. 2e).

### Establishment of PTX-resistant MKN45 cell line

**Figure 3:**
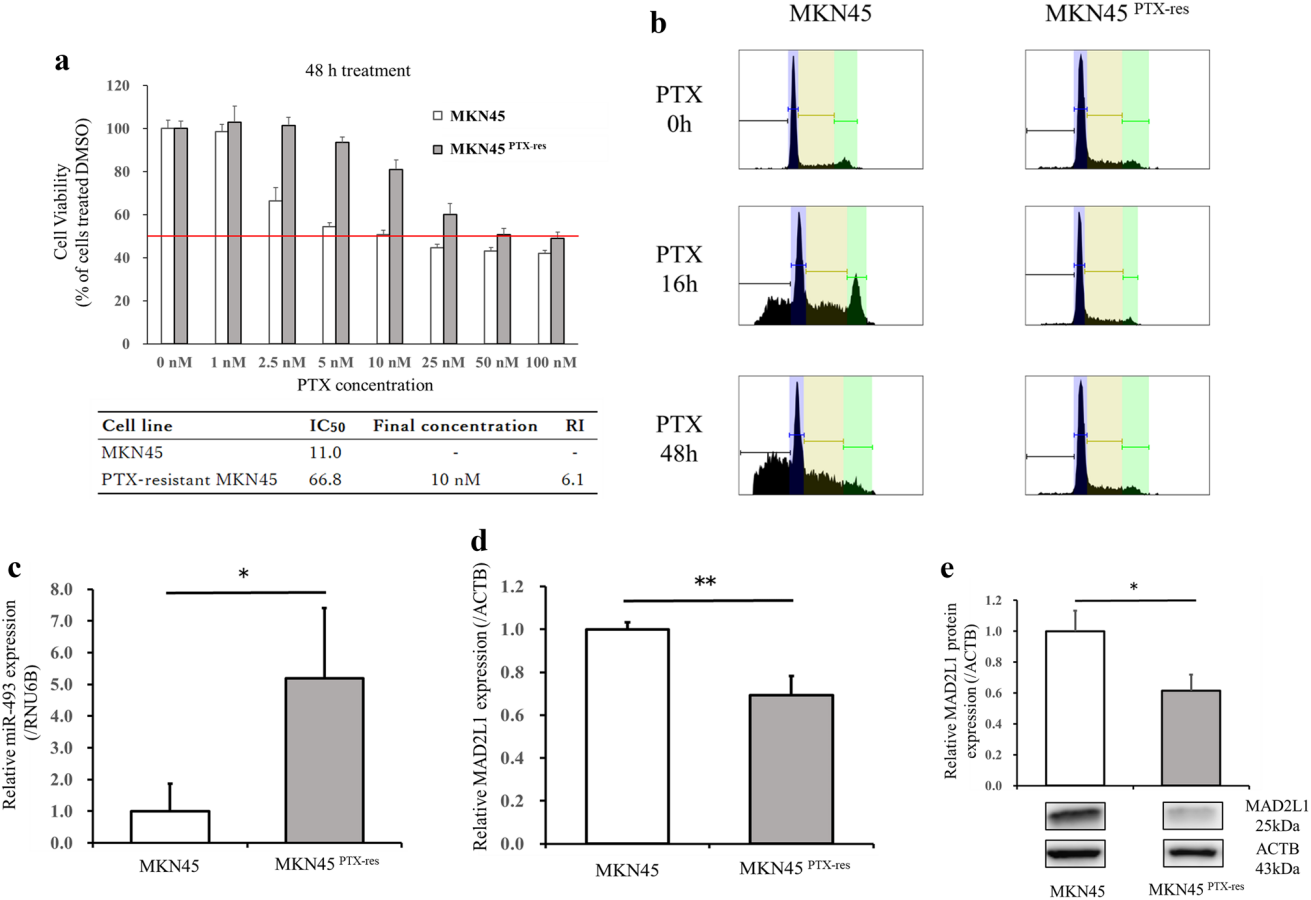
Establishment of a PTX-resistant MKN45 cell line. (**a**) Comparison of the IC_50_ values between the parental and MKN45^PTX-res^ cell lines. (**b**) Effect of PTX on the cell cycle of MKN45 and MKN45^PTX-res^ cells (**c**) miR-493 expression levels (**d**) MAD2L1 gene, and (**e**) protein expression levels in MKN45^PTX-res^. Statistical differences were determined using a Student’s t-test (*p < 0.05, **p < 0.005, ***p < 0.001).

We established a PTX-resistant MKN45 (MKN45^PTX-res^) cell line to elucidate the mechanism of acquired PTX resistance by comparison with the parental cell line. The MKN45^PTX-res^ cell line was established by an incremental, stepwise exposure to PTX. The PTX IC_50_ value for MKN45 was 11.0 nM, and 66.8 nM for MKN45^PTX-res^. The RI 6.1, exceeded the defined value and indicated that a resistant line had been established (Fig. 3a). To further assess the properties of the resistant cell line, a cell cycle analysis was performed. MKN45 and MKN45^PTX-res^ were cultured with 10 nM PTX for 0, 16, and 48 h. Neither cell line showed significant changes in the cell cycle after 0 h of treatment; however, the ratio of G2/M and subG1 phases increased after 16 h of treatment with MKN45. There was also a shift to the subG1 phase after 48 h of treatment, whereas no such change was observed in MKN45^PTX-res^ cells (Fig. 3b). The results suggest that MKN45^PTX-res^ has an impaired response to PTX by circumventing mitotic arrest and apoptosis.

### miR-493 suppresses MAD2L1 expression and reduces sensitivity to PTX of MKN45 cells

**Figure 4:**
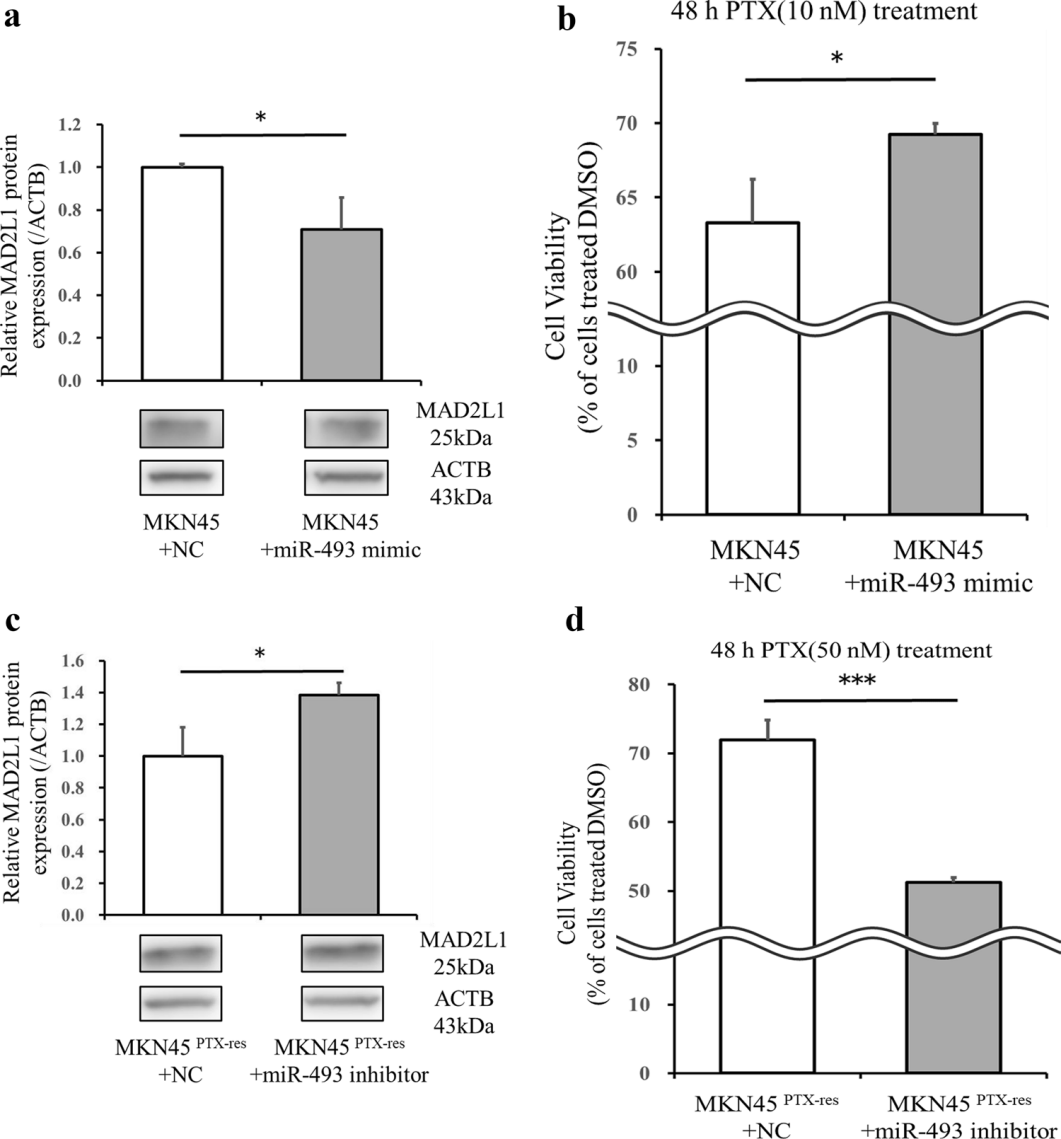
Effect of miR-493 expression on the sensitivity of MKN45 cells to PTX. (**a**) Comparison of MAD2L1 protein expression levels between miR-493 mimic-treated and control-treated MKN45 cells. A representative western blot is shown at the bottom. (**b**) Comparison of the sensitivity of miR-493 mimic-treated and control-treated MKN45 cells to PTX. (**c**) Comparison of MAD2L1 protein expression between miR-493 inhibitor-treated and control-treated MKN45^PTX-res^. A representative western blot is shown at the bottom. (**d**) Comparison of the sensitivity of miR-493 inhibitor-treated MKN45^PTX-res^ cells and control-treated MKN45^PTX-res^ cells to PTX. Statistical differences were determined using a Student’s *t*-test (*p < 0.05, **p < 0.005, ***p < 0.001).

To determine whether miR-493 suppresses MAD2L1 to sensitize MKN45 cells to PTX, we examined its expression in MKN45^PTX-res^ cells. The expression levels of miR-493 in MKN45^PTX-res^ cells were higher compared with that in MKN45 (Fig. 3c: *p* = 0.038). In addition, qRT-PCR and western blotting analysis revealed that MKN45^PTX-res^ cells exhibited lower MAD2L1 (Fig. 3d; *p* = 0.005) and protein (Fig. 3e, Supplementary Fig. S3; *p* = 0.017) expression compared with MKN45. Finally, we examined the effect of miR-493 on MAD2L1 expression by transfecting miR-493 mimics into MKN45 cells. Significantly downregulated levels of MAD2L1 protein were observed (Fig. 4a, Supplementary Fig. S4; *p* = 0.028) and reduced PTX sensitivity was evident (Fig. 4b; *p* = 0.023). Conversely, transfection of miR-493 inhibitor in MKN45^PTX-res^ cells resulted in increased MAD2L1 protein expression (Fig. 4c, Supplementary Fig. S5; *p* = 0.029), which restored PTX sensitivity (Fig. 4d; *p* = 0.001). These results suggested that miR-493 and MAD2L1 expression are involved in acquired PTX resistance in GC.

## Discussion

In this study, exosomes derived from the peritoneal fluid of GC patients with PM were cultured with MKN45 cells and cellular uptake was observed. MKN45 carrying exosomes from the CY + group were less sensitive to PTX compared with those carrying exosomes from the CY − group. Among the differentially expressed exo-miRs between the CY + and CY − groups, exo-miR-493 was selected and shown to be involved in acquired resistance to PTX through downregulated MAD2L1 expression.

It is unclear whether systemic administration of anticancer drugs is the appropriate route of administration for patients with PM. The Japanese Gastric Cancer Treatment Guidelines recommend systemic chemotherapy with platinum agents and fluoropyrimidine, plus trastuzumab or nivolumab depending on HER2 status, for unresectable or recurrent GC. PM is considered an unresectable/recurrent factor on par with liver metastasis and distant lymph node metastasis, and must be treated with systemic chemotherapy. The ATTRACTION-4 study was conducted to verify the effectiveness of oxaliplatin-based chemotherapy combined with nivolumab in patients with unresectable/recurrent GC. The results indicated efficacy in patients with liver and distant lymph node metastases, but it was unable to demonstrate efficacy in patients with PM^10^. One potential reason was the peritoneal-plasma barrier, which prevents intravenously administered drugs from reaching the peritoneal nodules as well as free cancer cells in the peritoneal cavity^26^. Therefore, it is reasonable to approach intraabdominal disease directly through the i.p. administration of anticancer drugs. Several clinical trials, including the PHOENIX-GC trial, have evaluated the efficacy of IP chemotherapy for carcinomas with peritoneal dissemination and have shown some efficacy^12,27^. For GC, the anticancer drug that has most often been administered intraperitoneally in patients with PM is PTX. It is hydrophobic and may be solubilized with Cremophor El^®^ for clinical use. Because of its relatively large formulation size when administered intraperitoneally, PTX does not readily diffuse into the blood, but is slowly absorbed via the peritoneal lymphatics from the abdominal cavity. This results in increased drug exposure to the intraabdominal region to enhance the anti-tumor effect^28–30^. To maintain a high concentration in the abdominal cavity, ICIs, which are antibody-based drugs with a high molecular weight, may be suitable for IP chemotherapy. Yamamoto et al. demonstrated in tumor-bearing mice that the amount of anti-PD-L1 antibody transferred into intraperitoneal tumors increased by approximately eight-fold following i.p. treatment compared with that administered intravenously, thus enhancing antitumor efficacy^31^. Taken together, i.p. administration is an attractive and efficient route of administration of anticancer drugs for patients with PM and warrants further study.

A second issue is the evaluation of treatment efficacy for patients with PM, which is usually done based on “Response evaluation criteria in solid tumors (RECIST)” guidelines^32^. However, unlike liver metastasis and distant lymph node metastasis cases, PM lacks measurable lesions, making it difficult to assess treatment efficacy, whereas the same is true for IP chemotherapy. Therefore, it is necessary to identify reliable biomarkers that can serve as indicators for the efficacy of IP chemotherapy at the beginning of treatment. In the present study, we validated exo-miRNA expression using a TaqMan miRNA assay. We compared expression between the CY + and CY − groups, which showed that exo-miR-493 may be a useful biomarker to predict the efficacy of IP chemotherapy. Recently, several studies have found that sequential chemotherapy after failure of first-line chemotherapy contributes to prolonged survival of advanced GC^33,34^. In addition, Hasegawa et al. suggested that integrating multiple clinicopathological parameters, such as clinical symptoms, tumor markers, and CT image results, to determine treatment efficacy and altering treatment contributes to an improved prognosis^35^. Nevertheless, it is difficult to use clinical symptoms, which are subjective, for evaluating disease progression in daily clinical practice. Therefore, considering treatment modification for patients with elevated exo-miR-493 at the beginning of treatment may contribute to prolonged prognosis when the patient is unsure whether to change treatment.

A third problem is identifying the underlying mechanisms responsible for the effectiveness of IP chemotherapy. In this study, we focused on MAD2L1, which induces PTX resistance. It is one of the key proteins that make up the spindle assembly checkpoint (SAC), which is a regulatory signaling pathway during mitosis^36^. The SAC prevents chromosome mis-segregation during mitosis, thereby preventing aneuploidy^37^, whereas PTX targets the SAC. Aneuploidy, which is a hallmark of aggressive solid tumors including GC^38,39^, results from SAC suppression and is closely related to carcinogenesis and malignant transformation^37,40^. The negative regulation of SAC function through decreased MAD2L1 is associated with acquired PTX resistance in GC cell lines^41^. In the present study, we demonstrated that decreased MAD2L1 expression in MKN45 cells contributes to decreased PTX sensitivity in vitro and we confirmed the relationship between low MAD2L1 expression and the negative effects of PTX on recurrent GC by immunostaining. Furthermore, RNA interference studies revealed that acquired drug resistance may occur through the negative regulation of MAD2L1 expression by miR-493. In latest recent study on Exo-miRNAs in ascites fluid, Jiaxin et al. demonstrated that exosomal hsa‑let‑7g‑3p and hsa‑miR‑10395‑3p derived from peritoneal lavage of GC with PM positively regulates TGFβ signaling by suppressing RAPGEF3 expression, which results in tumor proliferation and IP chemotherapy resistance^21^. Because of the high tumor heterogeneity of GC, our results are significant because they identified a novel pathway for the acquisition of IP chemotherapy resistance.

Some limitations to this study should be considered. First, clinical data are from a single center and the number of cases was small. The clinical relevance of exosomal miRNAs should be validated in a larger cohort. Second, only the MKN45 cell line was used in our in vitro experiments. It is unknown whether similar results would be obtained with other gastric cancer cell lines. Third, although our study identified MAD2L1 as a downstream effector, considering that the results of reproduction experiments of miR-493 suppression of MAD2L1 expression showed a small but significant difference, we cannot rule out the possibility that miR-493 functions through other target genes or signaling pathways. Therefore, the identification and validation of other targets are essential. In recent years, promising results have been reported on previously overlooked and newly discovered miRNAs. We only examined 380 known miRNAs, which were examined by a microarray kit, thus we may have missed other important miRNAs.

Reproduction experiments of miR-493 suppression of MAD2L1 expression showed significant differences, but did not show the expected marked suppression of expression.

## Conclusion

Intraperitoneal exo-miR-493 may be involved in PTX resistance through the downregulation of MAD1L1 in GC with PM. Exo-miR-493 may represent a useful biomarker for chemoresistance and prognosis of GC patients with PM, and may also be a promising therapeutic target.

### Supplementary Information

Supplementary Figures.

## Data Availability

The original contributions presented in the study are included in the article material. Further inquiries can be directed to the corresponding authors.
