# Supplementary material for: Exosomal miR-493 suppresses MAD2L1 and induces chemoresistance to intraperitoneal paclitaxel therapy in gastric cancer patients with peritoneal metastasis
Source: Sci Rep. 2024 May 2;14:10075. doi: 10.1038/s41598-024-60967-x (PMC11065888; doi:10.1038/s41598-024-60967-x)
Supplement: Supplementary file 1 — Supplementary Figures. [file 41598_2024_60967_MOESM1_ESM.pdf]

Exosomal miR-493 suppresses MAD2L1 and induces chemoresistance to intraperitoneal paclitaxel therapy in gastric cancer patients with peritoneal metastasis

Masahiro Makinoya, Kozo Miyatani, Yoshiaki Matsumi, Yu Sakano, Shota Shimizu, Yuji Shishido, Takehiko Hanaki, Kyoichi Kihara, Tomoyuki Matsunaga, Manabu Yamamoto, Naruo Tokuyasu, Shuichi Takano, Teruhisa Sakamoto, Toshimichi Hasegawa, Hiroaki Saito, Yuji Nakayama, Mitsuhiko Osaki, Futoshi Okada, and Yoshiyuki Fujiwara

Supplementary Figure S1. Comparison of miR-99a-5p, miR-193a-5p, miR-500 expression in exosomes between the CY+ and CY− groups ($p = 0.438$, $p = 0.415$, $p = 0.023$, respectively). For miR-362-5p and miR-523, the expression levels were not high enough to allow adequate comparisons. Statistical differences were determined using a Student’s t-test (* $p < 0.05$, ** $p < 0.005$, *** $p < 0.001$, n.s.: not significant).

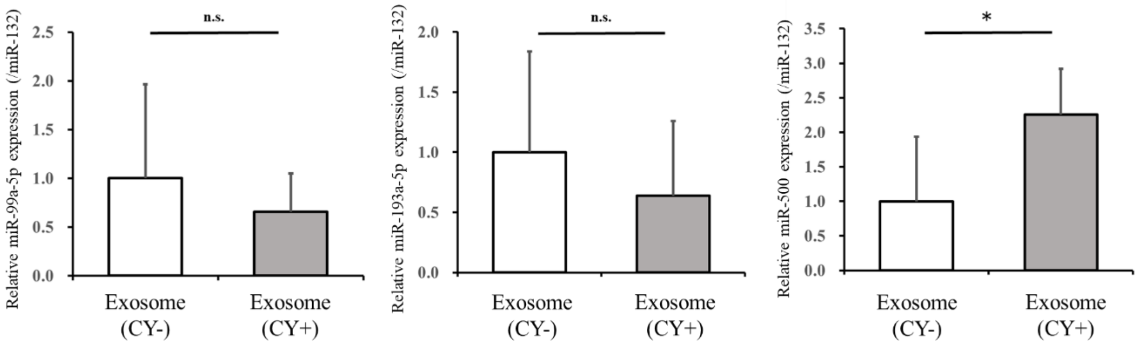

Supplementary Figure S2. MAD2L1 protein expression levels in MKN45 following siRNA knockdown of MAD2L1. The membrane was cut out of the region containing the band of interest with reference to the markers used as indicators that were simultaneously appliqued during electrophoresis.

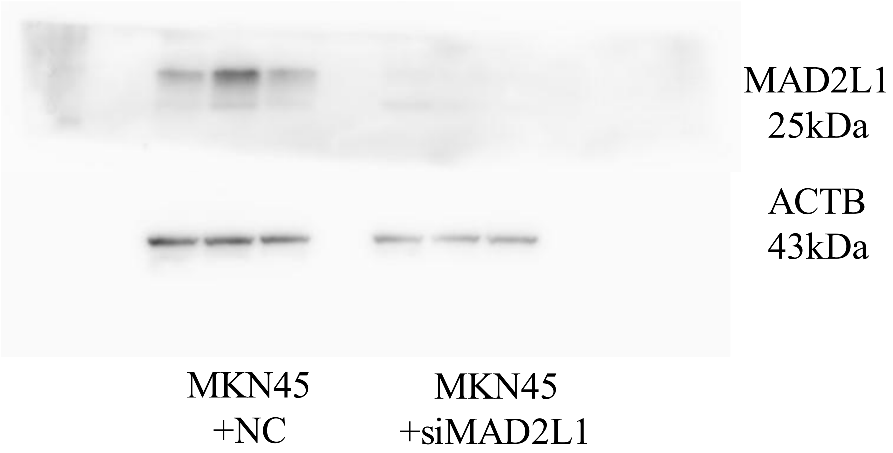

Supplementary Figure S3. MAD2L1 protein expression levels in MKN45$^{PTX\text{-}res}$.

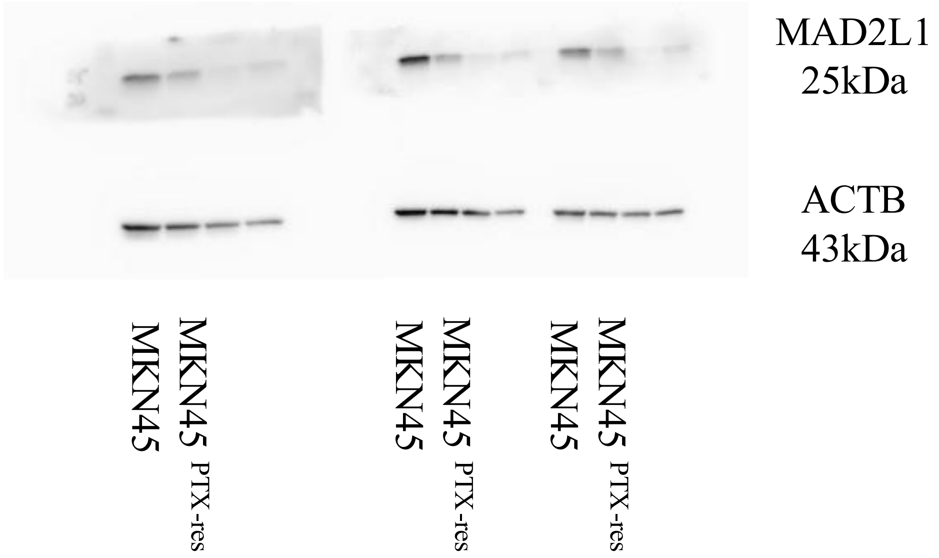

Supplementary Figure S4. Comparison of MAD2L1 protein expression levels between miR-493 mimic-treated and control-treated MKN45 cells. A representative western blot is shown at the bottom.

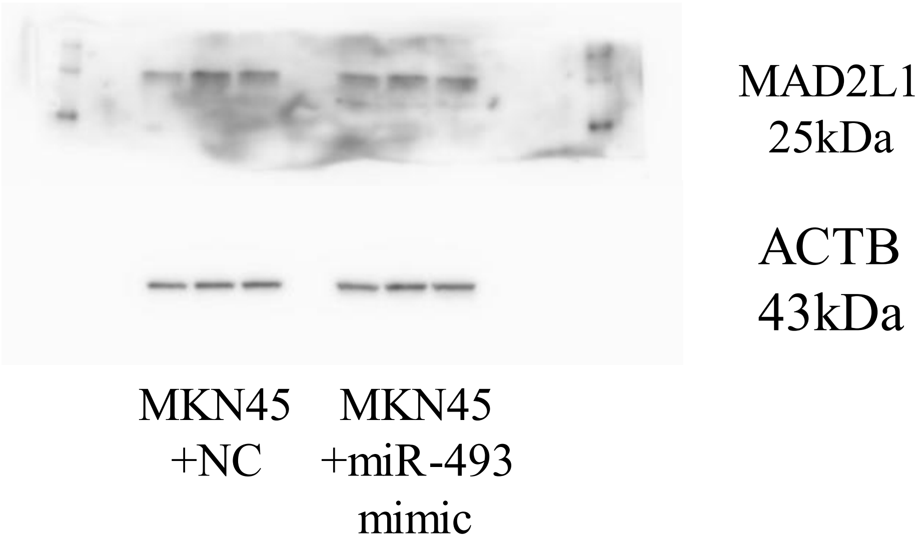

Supplementary Figure S5. Comparison of MAD2L1 protein expression between miR-493 inhibitor-treated and control-treated MKN45$^{PTX\text{-}res}$. A representative western blot is shown at the bottom.

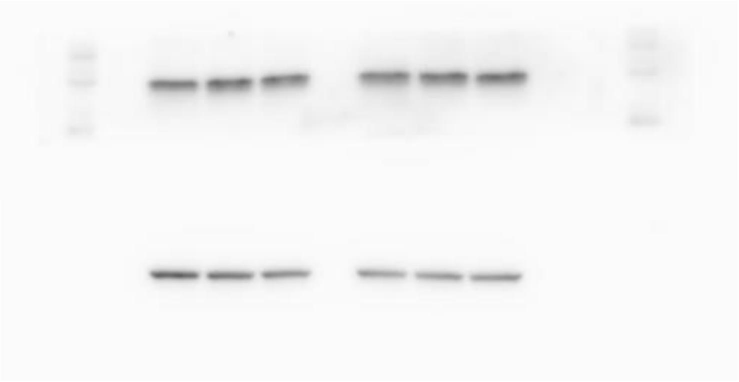

MAD2L1
25kDa

ACTB
43kDa

MKN45 $^{PTX\text{-}res}$
+NC

MKN45 $^{PTX\text{-}res}$
+miR-493
inhibitor
